# Disease progression in idiopathic pulmonary fibrosis with mild physiological impairment: analysis from the Australian IPF registry

**DOI:** 10.1186/s12890-018-0575-y

**Published:** 2018-01-25

**Authors:** Helen E. Jo, Ian Glaspole, Yuben Moodley, Sally Chapman, Samantha Ellis, Nicole Goh, Peter Hopkins, Greg Keir, Annabelle Mahar, Wendy Cooper, Paul Reynolds, E. Haydn Walters, Christopher Zappala, Christopher Grainge, Heather Allan, Sacha Macansh, Tamera J. Corte

**Affiliations:** 1 Royal Prince Alfred Hospital, Missenden Road, Camperdown, NSW 2050 Australia; 2 University of Sydney, Camperdown, NSW Australia; 3 The Alfred Hospital, Melbourne, VIC Australia; 4 Fiona Stanley Hospital, Murdoch, WA Australia; 5 Royal Adelaide Hospital, Adelaide, SA Australia; 6 The Austin Hospital, Heidelberg, VIC Australia; 7 Prince Charles Hospital, Chermside West, QLD Australia; 8 Princess Alexandria Hospital, Woolloongabba, QLD Australia; 9 School of Medicine, Western Sydney University, Parramatta, NSW Australia; 10 School of Medicine, University of Tasmania, Hobart, TAS Australia; 11 University of Tasmania, Hobart, TAS Australia; 12 Royal Brisbane and Women’s Hospital, Herston, QLD Australia; 13 John Hunter Hospital, New Lambton Heights, NSW Australia; 14 Lung Foundation Australia, Milton, QLD Australia

**Keywords:** Idiopathic pulmonary fibrosis, Interstitial lung disease, Disease severity

## Abstract

**Background:**

Idiopathic pulmonary fibrosis (IPF) is a progressive and fatal fibrosing lung disease of unknown cause. The advent of anti-fibrotic medications known to slow disease progression has revolutionised IPF management in recent years. However, little is known about the natural history of IPF patients with mild physiological impairment. We aimed to assess the natural history of these patients using data from the Australian IPF Registry (AIPFR).

**Methods:**

Using our cohort of real-world IPF patients, we compared FVC criteria for mild physiological impairment (FVC ≥ 80%) against other proposed criteria: DLco ≥ 55%; CPI ≤40 and GAP stage 1 with regards agreement in classification and relationship with disease outcomes. Within the mild cohort (FVC ≥ 80%), we also explored markers associated with poorer prognosis at 12 months.

**Results:**

Of the 416 AIPFR patients (mean age 70.4 years, 70% male), 216 (52%) were classified as ‘mild’ using FVC ≥ 80%. There was only modest agreement between FVC and DLco (k = 0.30), with better agreement with GAP (k = 0.50) and CPI (k = 0.48). Patients who were mild had longer survival, regardless of how mild physiologic impairment was defined. There was, however, no difference in the annual decline in FVC% predicted between mild and moderate-severe groups (for all proposed criteria). For patients with mild impairment (*n* = 216, FVC ≥ 80%), the strongest predictor of outcomes at 12 months was oxygen desaturation on a 6 min walk test.

**Conclusion:**

IPF patients with mild physiological impairment have better survival than patients with moderate-severe disease. Their overall rate of disease progression however, is comparable, suggesting that they are simply at different points in the natural history of IPF disease.

**Electronic supplementary material:**

The online version of this article (10.1186/s12890-018-0575-y) contains supplementary material, which is available to authorized users.

## Background

Idiopathic pulmonary fibrosis (IPF), a progressive fibrosing interstitial lung disease (ILD) of unknown cause, results in respiratory failure and ultimately death. Historically the median survival is thought to be only 2-5 years [1], and the incidence of IPF appears to be increasing globally [2]. Recently, two agents – nintedanib and pirfenidone – have demonstrated efficacy in slowing the rate of FVC decline as well as impacting on a number of other important disease outcomes [3–6]. However, each trial focused on cohorts with moderate physiologic impairment and the ASCEND trial excluded subjects with a forced vital capacity (FVC) above 90% predicted [3]. Although post-hoc analyses demonstrate comparable responses to anti-fibrotic therapy between participants with preserved lung volumes and those with more significant impairment, access to anti-fibrotic medications has been excluded from patients with lesser physiological impairment in several drug jurisdictions due to the limited nature of efficacy data for this cohort [7, 8].

There have been various attempts to define severity of IPF [9–11]. Physiologic measurements, such as FVC or diffusion capacity for carbon monoxide (DLco) have been commonly used [12, 13], as have composite measures of several physiologic values or demographic features [14, 15]. The two most commonly used composite scores are the composite physiological index (CPI) [14] and the GAP (gender, age and physiology) stage [15]. While both individual physiologic measurements and composite scores predict mortality, the ability of each to distinguish differences in disease progression among those with mild disease from those more significantly affected remains unknown. Clinically, the FVC has been used not only as a clinical trial endpoint, but also by funding authorities worldwide to limit access to anti-fibrotic medications.

The Australian IPF Registry (AIPFR), launched in 2012, recruits IPF patients from across Australia and a broad spectrum of impairment exists within its data set. Using this cohort, we sought to compare the FVC ≥ 80% with other severity criteria in their ability to distinguish differences in death and disease progression as measured by annual FVC decline, between mild and moderate-severe cohorts. Additionally, we investigated factors predicting poorer prognosis for patients with mild physiological impairment, to ascertain whether further characterisation may help select patients who would especially merit early drug intervention.

## Methods

### Participants

The Australian IPF Registry is a multi-centre, prospective, observational registry of incident and prevalent IPF patients across Australia. Details regarding the Australian IPF Registry data collection, structure and operations have previously been published [16]. The Registry has ethical approval to operate in all States and Territories and this analysis has ethical approval from the Sydney Local Health District ethics committee (protocol No X14–0264). All participants in the Registry were eligible for inclusion in this analysis.

### Classification of mild disease

Using data from the Registry, patients were categorised as having mild physiological impairment using four different criteria. The primary analysis focused on FVC ≥ 80% predicted (exploratory analysis for FVC ≥ 90% FVC ≥ 75%, FVC ≥ 70%) [13], as this is the criteria used clinically to restrict anti-fibrotic therapies in many regions. Secondary analysis included: DLco ≥55% (exploratory analysis of DLco ≥50%, DLco ≥40%, DLco ≥30%) [12]; CPI ≤ 40 (exploratory analysis CPI ≤ 30, CPI ≤ 50) [14]; GAP stage 1 [15].

Only patients who had all the necessary information for calculation of all scores were included. The CPI was calculated using the formula: CPI = 91.0 - (0.65 × % predicted DLco) - (0.53 × % predicted FVC) + (0.34 × % predicted FEV_1_) [14]. The GAP score was calculated using gender, age and physiology as outlined in Additional file 1: Table S1 [15].

### Statistical methods

Data are presented as mean ± standard deviation (SD) and also range, or n (%), as appropriate. The agreement between the different criteria used to define mild impairment was assessed using kappa values. Univariable and multivariable Cox proportional models were performed to investigate the relationships between baseline variables, including disease severity classifications, with survival. All patients who received lung transplantation were censored at the date of transplant for time to event analyses. Survival curves were compared using the Kaplan-Meier method and log rank test.

An unstructured, linear mixed model for changes in FVC % predicted per year was fitted with random intercepts and slopes to compare the disease trajectory for patients with mild compared with moderate-severe physiological impairment, over the entire time of patient follow up in the Registry. Finally, logistic regression assessing a composite end-point of death or disease progression (defined as a relative fall in FVC ≥ 10% and/or DLco ≥ 15% from baseline) at 12 months was performed in patients with mild impairment (FVC ≥ 80%) at baseline, to determine markers associated with poorer short-term prognosis in this group.

## Results

### Baseline characteristics

Of the 647 patients in the Australian IPF Registry, there were 416 patients who had complete data for severity score measurements at baseline, and so available for inclusion in this study. Their baseline characteristics are displayed in Table 1. Interestingly, though less severe, patients with mild physiological impairment still had significant symptoms as measured by the St George Respiratory Questionnaire (SGRQ) and University of California San Diego shortness of breath Questionnaire (UCSD SOBQ). Comparisons of baseline demographic parameters of patients excluded from this analysis are available in Additional file 2: Table S2. Overall, patients who were excluded were slightly older and had more severe symptoms, similar to patients categorised as severe impairment using FVC ≤ 80% criteria (Table 1).

**Table 1:** Baseline characteristics of patients in the Australian IPF Registry

Variable	n	overall	Mild (FVC ≥ 80%)	Moderate-Severe (FVC < 80%)	P^a^	Min	Max
			216	200			
Age, years	416	70.4 (8.6)	71.6 (7.9)	69.0 (9.1)	0.003	31.5	89.9
Male, n (%)	416	290 (69.7%)	125 (57.9%)	165 (82.5%)	<0.001		
Ever smoker, n (%)	416	308 (74.0%)	160 (74%)	148 (74%)	0.986		
BMI, kg/m^2^	412	28.8 (4.8)	28.9 (5.0)	28.6 (4.6)	0.565	15.8	46.4
FVC, L	416	2.6 (0.8)	2.96 (0.79)	2.30 (0.54)	<0.001	0.9	4.8
FVC, % pred	416	81.7 (21.2)	97.3 (16.6)	64.9 (9.8)	<0.001	40.9	219.5
FEV_1_/FVC ratio%	416	82.2 (8.4)	80.1 (8.6)	84.4 (7.5)	<0.001	27.0	135.0
DLco, % pred	416	48.5 (16.8)	54.6 (17.4)	41.8 (13.2)	<0.001	9.4%	143.7%
CPI	416	45.1 (14.1)	37.5 (13.2)	53.3 (9.7)	<0.001	54.2	74.5
GAP stage	416				<0.001		
GAP stage 1		192 (46.2%)	152 (70.4%)	40 (20.0%)			
GAP stage 2		186 (44.7%)	64 (29.6%)	122 (61.0%)			
GAP stage 3		38 (9.1%)	0 (0%)	38 (19.0%)			
6MWT distance, m	164	431.0 (119)	433 (115)	430 (122)	0.894	48.0	706.0
Initial SpO_2,_ %	163	95.5 (3.19)	95.8 (3.4)	95.4 (3.1)	0.482	83.0	100.0
End SpO_2,_ %	160	86.2 (7.2)	89.0 (7.0)	84.4 (6.8)	<0.001	58.0	99.0
Nadir SpO_2,_ %	153	85.3 (7.1)	87.9 (7.3)	83.6(6.5)	<0.001	58.0	99.0
SGRQ	383	43.0 (19.7)	38.7 (19.0)	47.6 (44.8)	<0.001	0.0	96.6
UCSD SOBQ	243	40.8 (29.3)	33.7(27.1)	48.6 (29.6)	<0.001	0.0	119.0
Cough severity	347	39.5 (23.5)	36.9 (22.9)	42.3(23.9)	0.034	0.0	100.0
Anti-fibrotic therapy	416	102 (24.5%)	52 (24.1%)	50 (25.0%)	0.826		

^a^comparison between mild and severe physiological impairment

There were 114 deaths (27.4%) over a median follow up period of 2.09 years (IQR 1.56, 2.78 years) providing 914 patient-years of follow up. 17 (4.1%) patients underwent lung transplantation during the follow up period.

### Agreement of proposed criteria for mild IPF

**Fig. 1:**
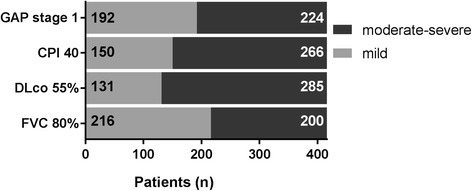
Classification as mild disease using different mild definitions

The FVC ≥ 80% categorised almost equal numbers of patients as having mild (*n* = 216, 52%) compared to moderate-severe impairment (*n* = 200, 48%), while a DLco ≥55% classified the least patients as having mild disease (*n* = 131, 32%) (Fig. 1). We also undertook exploratory analysis of other threshold levels and as expected, the change in threshold values had a significant impact on the proportion of patients classified as mild for FVC, DLco and in particular CPI (Additional file 3: Figure S1).

Mild physiological impairment defined by FVC ≥ 80% had modest agreement with GAP stage 1 (k = 0.50) and CPI ≤ 40 (k = 0.48) and only fair agreement with DLco ≥ 55% (k = 0.30). CPI ≤ 40 had the best agreement with other variables including DLco ≥ 55% (k = 0.74) and GAP stage 1 (k = 0.68). There was good agreement between GAP stage 1 and DLco ≥ 55% (k = 0.61).

### Factors predictive of death

As demonstrated in Table 2, on univariable analysis, demographic characteristics (age, gender, BMI, smoking status), physiology, and symptom scores (SGRQ, UCSD SOBQ), except cough severity (measured at baseline on a visual analogue scale), all predicted increased mortality for the entire IPF population.

**Table 2:** Univariable Cox analysis

	n	HR	95% CI		p
Age	416	1.03	1.01	1.05	0.013
male	416	1.96	1.25	3.08	0.003
BMI	412	0.94	0.89	0.98	0.003
Smoking	416	1.93	1.20	3.11	0.007
Physiology					
FVC (L)	416	0.56	0.43	0.74	<0.001
FVC (%pred)^a^	416	0.71	0.64	0.79	<0.001
DLco (%pred)^a^	416	0.56	0.49	0.64	<0.001
6MWD^b^	164	0.82	0.73	0.92	0.001
SpO2 at rest	163	0.88	0.82	0.95	0.001
SpO2 at end	160	0.92	0.88	0.95	<0.001
SpO2 nadir	153	0.92	0.89	0.96	<0.001
CPI	416	1.09	1.07	1.11	<0.001
GAP stage	416	4.48	3.37	5.95	<0.001
Patient reported outcomes					
SGRQ total^c^	383	1.11	1.06	1.15	<0.001
UCSD SOBQ^a^	243	1.23	1.13	1.33	<0.001
Cough severity^a^	347	1.08	0.99	1.17	0.069

*BMI* body mass index, *FVC* forced vital capacity, *DLco* diffusion capacity for carbon monoxide, *CPI* composite physiological index, *GAP* gender age physiology, 6*MWD* 6 min walk distance, *SpO*2 peripheral blood oxygen saturation, *SGRQ* St George’s Respiratory Questionniare, *UCSD- SOBQ* University of California San Diego shortness of breath questionnaire. ^a^For every 10 unit change; ^b^ for every 50 m change; ^c^for every 4 point change

**Fig. 2:**
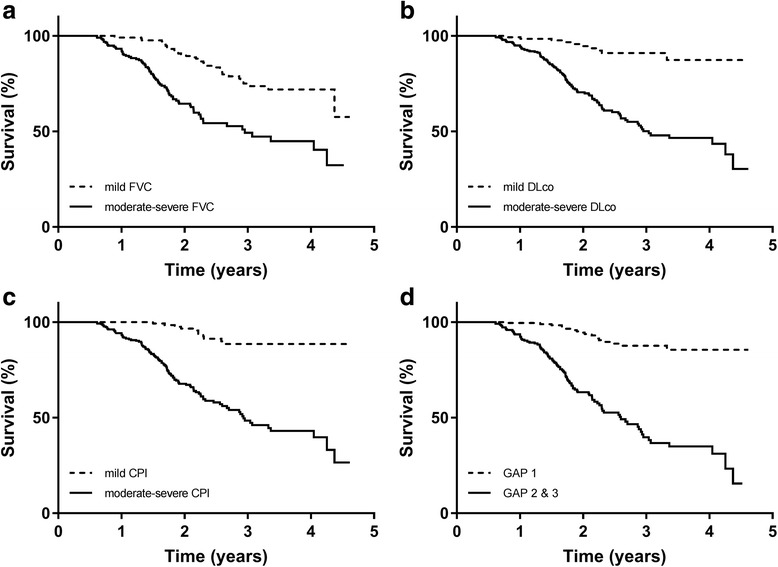
Kaplan Meier analysis for time to death by disease category. **a**. Kaplan Meier analysis according to FVC 80%. Log rank test *p* < 0.001. **b**. Kaplan Meier analysis according to DLco 55%. Log rank test p < 0.001. **c**. Kaplan Meier analysis according to CPI 40. CPI (composite physiological index). Log rank test p < 0.001. **d**. Kaplan Meier analysis according to GAP stage. GAP (Gender, Age, Physiology). Log rank test p < 0.001

The presence of mild impairment at baseline (by all proposed criteria) was also predictive of better survival than those patients with moderate-severe disease, on univariable as well as multivariable analysis including age, gender, BMI and smoking status (Table 3). There was a wide separation in the Kaplan-Meier curves for mild versus moderate-severe physiological impairment (Fig. 2).

**Table 3:** Univariable and multivariable Cox analysis for disease severity

	HR	95% CI	p	HR	95% CI	p
	Univariable analysis			Multivariable analysis^a^		
FVC < 80%	3.21	2.17–4.75	<0.001	3.15	2.11–4.71	<0.001
DLco < 55%	6.28	3.28–12.03	<0.001	5.55	2.88–10.70	<0.001
CPI > 40	7.28	3.90–13.57	<0.001	6.60	3.52–12.39	<0.001
GAP stage 1	7.09	4.27–11.76	<0.001	7.44	4.18–13.25	<0.001

^a^Multivariable model includes age, gender, BMI and smoking status and includes 412 of 416 available for univariable model

### Disease progression

**Fig. 3:**
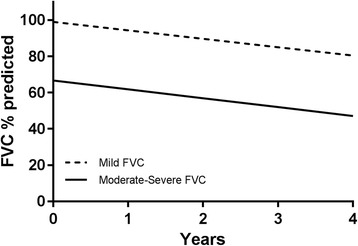
Decline in FVC % predicted in mild (FVC ≥ 80%) compared with moderate-severe (FVC < 80%) physiological impairment. Graphical representation of annual FVC% decline as calculated by unstructured linear mixed model with random intercept and slopes for mild and moderate-severe disease

The disease progression of patients with mild impairment, measured as change in FVC % predicted per year compared to those with moderate-severe impairment are shown in Table 4 and Fig. 3. There was no difference in the annual rate of FVC% predicted decline between the mild and moderate-severe cohorts, regardless of the disease severity classification used, with the predicted annual fall for the entire cohort calculated at 4.4 units (of FVC % predicted)/year. In a exploratory analysis, locally weighted scatterplots of FVC% predicted were constructed for patients with mild and moderate-severe disease and again demonstrated similar disease trajectories in these groups (Additional file 4: Figure S2).

**Table 4:** Annual FVC % predicted decline by disease category and severity

	mild	95% CI	more severe	95% CI	P^a^
FVC	−4.6%	−5.8;-3.4%	−4.9%	−6.3; −3.5%	0.779
DLco	−4.9%	−6.0;-3.8%	−4.7%	−5.7; −3.7%	0.702
CPI	−5.0%	−6.3; −3.6%	−4.5%	−5.7; −3.3%	0.600
GAP	−5.0%	−6.3; −3.8%	−4.4%	−5.8; −3.0%	0.517

^a^comparison of mild to moderate-severe group

### Factors predicting progression or death at 12 month in the mild disease group

There were 216 patients with FVC ≥ 80% categorised as having mild impairment. Overall, these patients had preserved lung volume with a mean FVC of 97.3% predicted, DLco of 54.6% predicted and were mostly categorised as GAP stage 1 (70.4%) with the remainder as GAP stage 2 (29.6%). Unsurprisingly, they were less symptomatic than those with more severe disease, though they still had significant symptoms. Interestingly, patients with mild impairment were slightly older and more likely to be female (Table 1).

Within the cohort of patients with mild impairment, the prognostic markers which predicted death or progression at 12 months were evaluated using univariable logistic regression. There were 22 patients (10.2%) who died or progressed within 12 months. The only factor that predicted death or progression at 12 months was oxygen desaturation during 6 min walk testing at baseline (OR 0.89, 95% CI 0.81, 0.98; *p* = 0.024 for every 1% change in nadir SpO_2_) (Table 5).

**Table 5:** Logistic regression for death or disease progression at 12 months in patients with FVC ≥ 80%

	n	OR	95% CI		p
Age	216	0.99	0.94	1.05	0.847
male	216	0.86	0.35	2.09	0.739
BMI	212	1.00	0.92	1.10	0.954
Ever smoker	216	0.93	0.34	2.50	0.879
FVC % pred^a^	216	0.81	0.56	1.16	0.241
DLco %pred^a^	216	0.87	0.67	1.14	0.312
CPI^a^	216	1.26	0.86	1.86	0.234
GAP stage	216	1.12	0.43	2.90	0.813
6 MWD^b^	63	0.75	0.54	1.04	0.083
SpO2 at start	63	0.86	0.71	1.03	0.108
SpO2 at end	63	0.89	0.81	0.98	0.021
SpO2 nadir	59	0.89	0.81	0.98	0.024
SGRQ^c^	199	1.02	0.93	1.12	0.688
UCSD SOBQ^a^	126	1.00	0.77	1.28	0.970
Cough severity^a^	177	1.16	0.95	1.41	0.136

*BMI* body mass index, *FVC* forced vital capacity, *DLco* diffusion capacity for carbon monoxide, *CPI* composite physiological index, *GAP* gender age physiology, 6*MWD* 6 min walk distance, *SpO*2 peripheral blood oxygen saturation, *SGRQ* St George’s Respiratory Questionniare, *UCSD- SOBQ* University of California San Diego shortness of breath questionnaire. ^a^For every 10 unit change; ^b^ for every 50 m change; ^c^ for every 4 point change

Unsurprisingly, within this cohort with mild impairment, other lung function parameters and composite scores were not predictive of death or progression. On a multivariable logistic regression model including age, gender, BMI and smoking status, nadir oxygen saturation during 6MWT remained a significant predictor of poorer outcome (OR 0.89, 95%CI 0.80, 0.98; p = 0.024).

## Discussion

In our cohort of IPF patients from the Australian IPF Registry, we demonstrate that a significant proportion of IPF patients have mild physiological impairment; though this proportion varied depending on the criteria and threshold used to define mild impairment. We demonstrate that the agreement for mild impairment using FVC ≥ 80% was modest to fair with other proposed criteria. We also show that patients who are mild have better survival than patients those with moderate-severe physiological impairment. Importantly, we show that patients with mild impairment, regardless of the criteria for categorisation used, have a similar annual rate of decline in FVC % predicted compared to those with more severe impairment. Finally, we show that in patients with mild impairment, the prognostic marker most predictive of death or progression at 12 months was oxygen desaturation on the 6 min walk test (6MWT).

One important finding of this study was that IPF patients with mild physiological impairment had the same disease trajectory, with comparable annual decline in FVC to those with moderate-severe disease. This mirrors the results found in the post-hoc analysis of pirfenidone [8] and nintedanib trial cohorts [7] whereby patients with mild physiological impairment were shown to have comparable disease progression at 12 months to their more severe counterparts. Unlike the clinical trial cohorts however, Registry patients are real-world, unselected IPF patients with frequent significant co-morbidities. Registry patients also have a wide spectrum of IPF disease severity with 68 patients below the threshold for inclusion in clinical trials (FVC < 50%, *n* = 9; DLco <30% *n* = 47, and *n* = 6 for both), and are managed by respiratory physicians throughout Australia, not limited to tertiary referral centres. The reproducibility of this finding in this heterogeneous cohort strengthens the generalisability of the post-hoc analysis from the large therapeutic clinical trials [7, 8]. It is also important to note that even patients with mild physiological impairment had significant symptoms. Ultimately, these results suggest that patients with preserved physiology are likely to represent an earlier stage of the natural history of IPF, rather than a separate and milder cohort of IPF patients with a specific natural history.

Another important finding of our study was that within the cohort of patients with mild impairment, the degree of oxygen desaturation on the 6MWT was the only significant short-term prognostic marker. Whilst previous studies have demonstrated that the 6MWT distance [17–19] and oxygen desaturation [20, 21] on 6MWT can predict mortality in IPF, this test is often not performed on a regular basis (as demonstrated in this study by the smaller number of patients who have had this test). The desaturation on the 6MWT is likely to represent a complex interplay between, and cumulative impact of, the physiological changes that occur in IPF including pulmonary hypertension, ventilation/perfusion mismatch and diffusion limitation. Our data suggests that in a cohort with relatively preserved lung function, where individual pulmonary function variables provide no additional prognostic value, the performance of the 6MWT is prognostically important.

We also highlight the differences between various proposed criteria for IPF severity, reflecting the need for a standardised approach to disease severity stratification. While we found in our cohort a significant overlap using these various definitions for mild physiological impairment, there were also significant discrepancies. Furthermore, there was only fair concordance between FVC and DLco, with the FVC ≥80% classifying more patients as mild than DLco ≥55%. This likely reflects the effects of the pulmonary function thresholds chosen, and was highlighted by the considerable variability in classification with different thresholds used in the exploratory analysis (see Additional file 3). The better concordance with composite values, as opposed to between single measures such as FVC and DLco, arises from the fact that both these single measures contribute to the calculation of the composite scores. It is interesting to note that the DLco and the composite scores were better at predicting survival suggesting that, while it is commonly used, the FVC threshold may not be the most clinically useful criteria.

Our study has several limitations. Firstly, this was a retrospective analysis of a prospective observational study and all investigations were performed as part of the patients’ routine clinical care, resulting in some variability in the investigations performed. In particular, in clinical practice in Australia, there is variability between centres in routinely undertaking a 6MWT, for which we had baseline data for 164 individuals overall, and 63 patients in the groups with FVC ≥ 80%. Despite the limited numbers, we were able to show a significant difference in outcomes, highlighting the strength of this association, a finding that we hope will now affect national clinical practice. Also, while there is a standardised guide for the 6MWT, there is likely to be variation between centres. The potential importance of this association between desaturation on 6MWT and prognosis also highlights the importance of confirming these findings in a larger, prospective cohort with standardised testing.

## Conclusion

In summary, accurate clinical staging has important implications in IPF as it can inform initial patient management, prognostication and standardisation of clinical trials and their outcomes. Patients with mild physiological impairment have better overall survival, although their rate of disease progression appears the same as seen in those with moderate-severe impairment, with differences in outcome being attributable to where the patient sits in the natural history of their disease. Given their apparent identical disease trajectory and significant symptoms, this study adds impetus to the view that patients with mild impairment be offered anti-fibrotic therapy early in their disease course. This is particularly so for those with significant desaturation on the 6MWT, as they appear to have the poorest outcomes.

## Additional files

Additional file 1: Table S1. GAP stage calculation. (DOC 30 kb)

Additional file 2: Table S2. Baseline characteristics of patients excluded from analysis. (DOC 35 kb)

Additional file 3: Figure S1. Exploratory analysis of varying thresholds. (PNG 100 kb)

Additional file 4: Figure S2. Locally Weighted Scatterplot Smoothing curves for FVC% predicted. a. Mild physiological impairment (FVC ≥ 80%). b. Moderate-severe physiological impairment (FVC < 80%). c. Summary of LOWESS curve means. (ZIP 212 kb)

## Abbreviations

6MWD: 6 min walk distance
6MWT: 6 min walk test
AIPFR: Australian Idiopathic Pulmonary Fibrosis Registry
ASCEND: Assessment of Pirfenidone to Confirm Efficacy and Safety in Idiopathic Pulmonary Fibrosis
BMI: body mass index
CAPACITY: Clinical Studies Assessing Pirfenidone in idiopathic pulmonary fibrosis: Research of Efficacy and Safety Outcomes
COPD: chronic obstructive pulmonary disease
CPI: composite physiological index
DLco: diffusion capacity for carbon monoxide
FVC: forced vital capacity
GAP: Gender, Age, Physiology
ILD: interstitial lung disease
IPF: idiopathic pulmonary fibrosis
NHS: National Health Service
SD: standard deviation
SGRQ: St George’s Respiratory Questionnaire
SpO2: peripheral blood oxygen saturation
TOMORROW: To improve pulmonary fibrosis with BIBF 1120
UCSD SOBQ: University of California San Diego shortness of breath questionnaire
