# Supplementary material for: Disease progression in idiopathic pulmonary fibrosis with mild physiological impairment: analysis from the Australian IPF registry
Source: BMC Pulm Med. 2018 Jan 25;18:19. doi: 10.1186/s12890-018-0575-y (PMC5785886; doi:10.1186/s12890-018-0575-y)
Supplement: Supplementary file 1 — GAP stage calculation. (DOC 30 kb) [file 12890_2018_575_MOESM1_ESM.doc]

Additional file 1: Table S1. GAP stage calculation

|  | **Predictor** | **Points** |
| --- | --- | --- |
| **G** | **Gender**  Female  Male | 0  1 |
| **A** | **Age, y**  ≤60  61-65  >65 | 0  1  2 |
| **P** | **Physiology**  **FVC % predicted**  >75  50-75  <50  **DLco, % predicted**  >55  36-55  ≤35  Cannot perform* | 0  1  2  0  1  2  3 |

*No patients were scored as “cannot perform” in this analysis, as any patient without a DLco measurement was excluded from the whole analysis.

| GAP stage | Points |
| --- | --- |
| Stage 1 | 0-3 |
| Stage 2 | 4-5 |
| Stage 3 | 6-8 |
