# Supplementary material for: Disease progression in idiopathic pulmonary fibrosis with mild physiological impairment: analysis from the Australian IPF registry
Source: BMC Pulm Med. 2018 Jan 25;18:19. doi: 10.1186/s12890-018-0575-y (PMC5785886; doi:10.1186/s12890-018-0575-y)
Supplement: Supplementary file 2 — Baseline characteristics of patients excluded from analysis. (DOC 35 kb) [file 12890_2018_575_MOESM2_ESM.doc]

**Additional file 2: Table S2**.

| **Variable** | **Included** | **excluded** | **P** |
| --- | --- | --- | --- |
| n | 416 | 231 |  |
| **Age, years** | 70.4 | 71.8 | 0.036 |
| **Male, n (%)** | 290 (69.7%) | 148 (64.1%) | 0.462 |
| **Ever smoker, n (%)** | 308 (74.0%) | 175 (75.8%) | 0.630 |
| **BMI, kg/m^2^** | 28.8 | 27.8 | 0.150 |
| SGRQ | 43.0 | 47.1 | 0.023 |
| USOB | 40.8 | 48.1 | 0.032 |
| Cough severity | 39.5 | 40.4 | 0.699 |
