# Supplementary figures and images for: Disease progression in idiopathic pulmonary fibrosis with mild physiological impairment: analysis from the Australian IPF registry
Source: BMC Pulm Med. 2018 Jan 25;18:19. doi: 10.1186/s12890-018-0575-y (PMC5785886; doi:10.1186/s12890-018-0575-y)

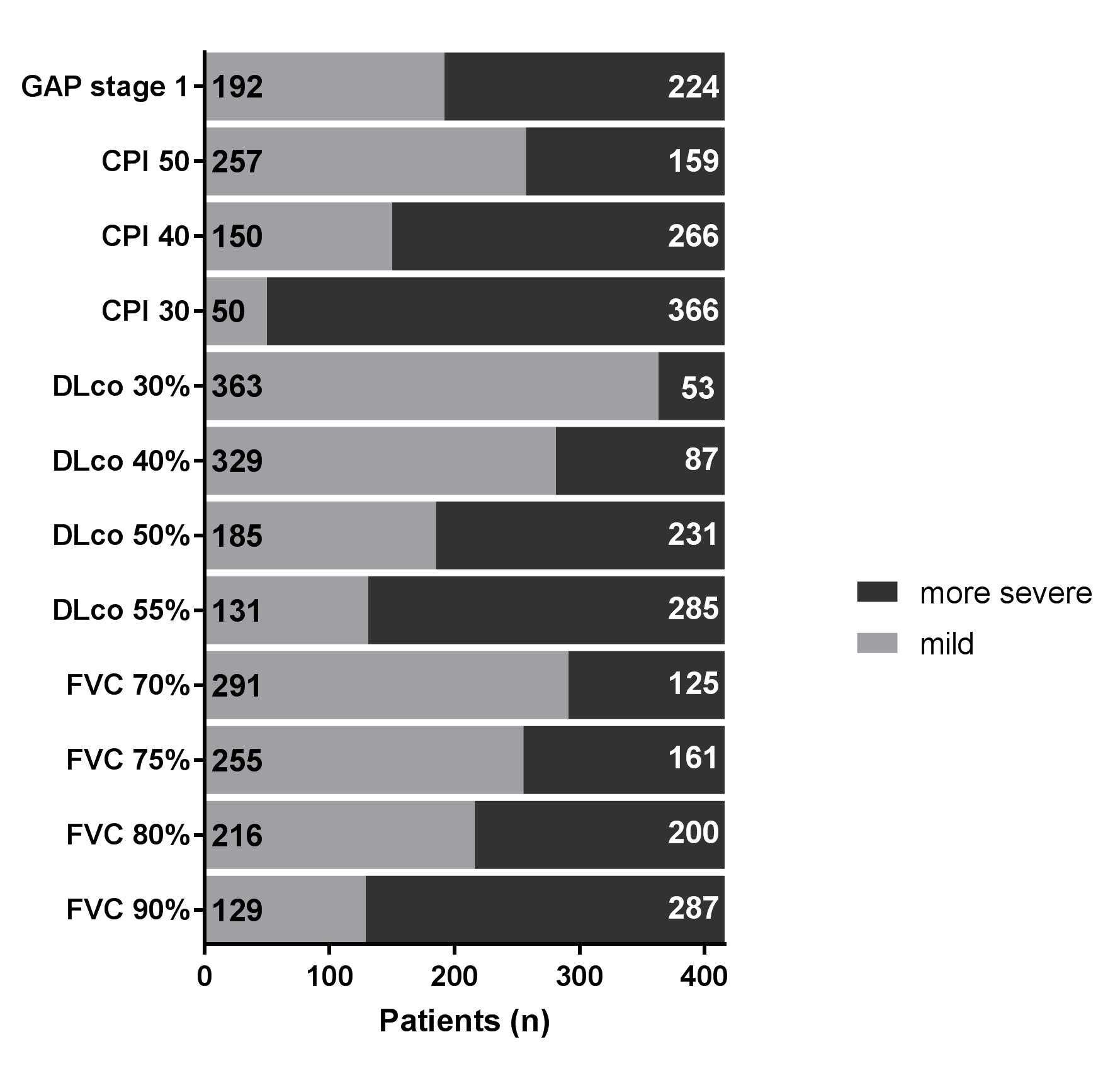

Supplement: Supplementary file 3 — Exploratory analysis of varying thresholds. (PNG 100 kb) [file 12890_2018_575_MOESM3_ESM.png]

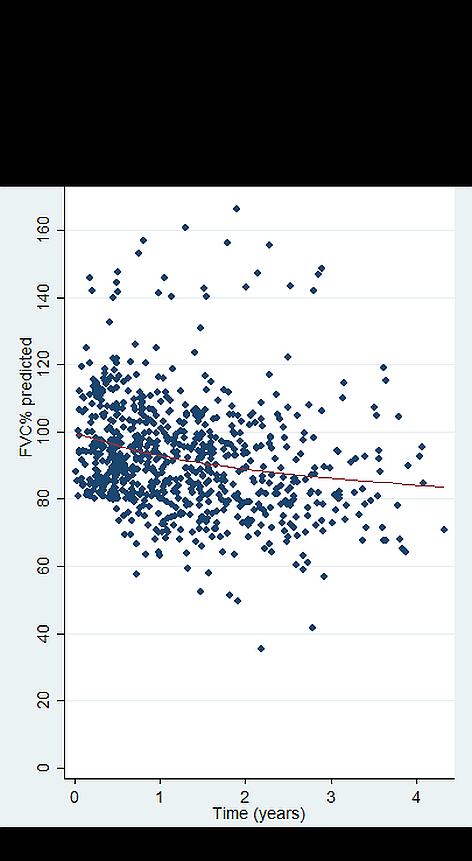

Supplement: Supplementary file 4 — Locally Weighted Scatterplot Smoothing curves for FVC% predicted. a. Mild physiological impairment (FVC ≥ 80%). b. Moderate-severe physiological impairment (FVC < 80%). c. Summary of LOWESS curve means. (ZIP 212 kb) [file 12890_2018_575_MOESM4_ESM.zip › supp figure 2a Mild LOWESSR3.tif]

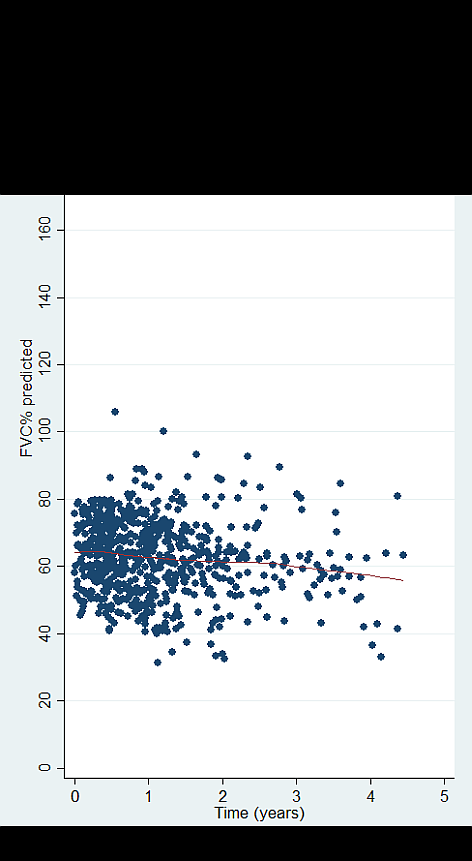

Supplement: Supplementary file 4 — Locally Weighted Scatterplot Smoothing curves for FVC% predicted. a. Mild physiological impairment (FVC ≥ 80%). b. Moderate-severe physiological impairment (FVC < 80%). c. Summary of LOWESS curve means. (ZIP 212 kb) [file 12890_2018_575_MOESM4_ESM.zip › supp Figure 2b Moderate-severe LOWESSR3.tif]

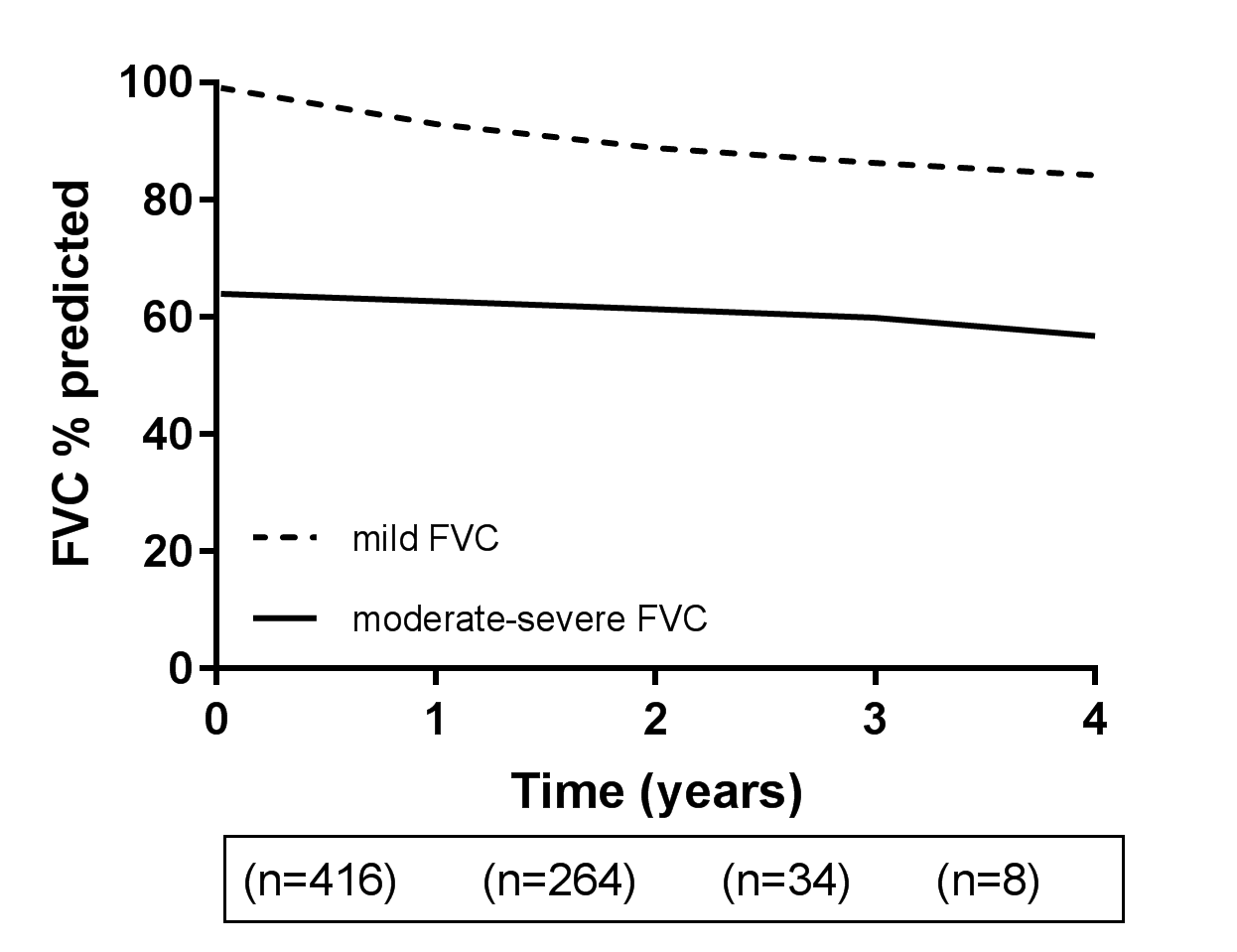

Supplement: Supplementary file 4 — Locally Weighted Scatterplot Smoothing curves for FVC% predicted. a. Mild physiological impairment (FVC ≥ 80%). b. Moderate-severe physiological impairment (FVC < 80%). c. Summary of LOWESS curve means. (ZIP 212 kb) [file 12890_2018_575_MOESM4_ESM.zip › supp figure 2cR3.tif]
